# Supplementary material for: Metabolomic Insights into COVID-19 Severity: A Scoping Review
Source: Metabolites. 2024 Nov 12;14(11):617. doi: 10.3390/metabo14110617 (PMC11596841; doi:10.3390/metabo14110617)
Supplement: Supplementary file 1 [file metabolites-14-00617-s001.zip › metabolites-3258774-SM-11.12.pdf]

SUPPLEMENTAL TABLE 2. Metabolites altered in COVID-19 cases across the 42 articles selected for COVID-19 severity.

Study	Metabolites altered		Statistical model	Sample size	Reference
	**Upregulated COVID19**	**Downregulated COVID19**			
1. 1H qNMR-Based Metabolomics Discrimination of Covid-19 Severity	**Covid-19 vs control**<br>Acetate, phenylalanine, leucine, lactate, glucuronate, creatine, glucose, glutamate, valine, tyrosine, tryptophan, pyruvate	**Covid-19 vs control**<br>glycerol, 3-aminoisobutyrate, threonine, citrate,	H-NMR, Untargeted, PCA and PLSDA	110	Correia B.S et. al. [1]
2. Targeted metabolomics identifies high performing diagnostic and prognostic biomarkers for COVID19	**COVID19 + vs COVID19 -**<br>LysoPC a C26:0, LysoPC a C28:0, kynurenine/tryp, propionic acid, kynurenine, phenylalanine, pyruvic acid, LysoPC a C26:1, LysoPC a C28:1, alpha-ketoglutarate,	**COVID19 + vs COVID19 -**<br>HPHPA, PC aa C32:2, tryptophan, p-hydroxypyuric acid, PC aa C36:0	LC-MS/MS, targeted, PLS-DA	161	Lopez-Hernandez Y, et al. [2]
3. Combining semi-targeted metabolomics and machine learning to identify metabolic alterations in the serum and urine of hospitalized patients with COVID19	**COVID19 vs No COVID-19**<br>Pentose phosphate, pentose and glucuronate interconversion, nucelotide sugars, ascorbate, and aldarate, cysteine, methionine, glycine, betaine, lauric acid, maltose, manonic acid, xylitol, etythronic acid, galacturonic acid, malic acid	**COVID19 vs No COVID-19**<br>S-adenosylhomocisteine, d-xylitol, maltose, fructose, glyceric acid, phosphoric acid, serum succinate	GC/MS with GC/Q-TOF, untargeted, LDA, Wilcoxon rank-sum test, FDR, ROC, and AUROC	171	Baiges-Gaya G, et al. [3]
4. Amino Acid Metabolism is Significantly Altered at the Time of Admission in Hospital for Severe	**COVID-19 acute phase vs recovery phase**<br>3-Hydroxy-DL-Kynurenine, carnitine, isovalerylcarnitine, kynurenine, acetylcarnitine, asparagine, glutamic acid, isoleucine, lysine, methionine, phenylalanine, threonine, tyrosine, valine, ornithine, taurine.	**COVID-19 acute phase vs recovery phase**<br>4-hydroxyproline, citrulline, l-glutamine, l-octanoylcarnitine, l-proline, l-tryptophan	LC-MS, targeted, PCA	71	Ansone L, et al. [4]

COVID-19 Patients: Findings from Longitudinal Targeted Metabolomics					
5. Longitudinal metabolomics of human plasma reveals prognostic markers of COVID-19 disease severity	**COVID + vs COVID-**<br>1-Methyladenosine, Cer_NS d18:1_16:0, Cer_NSd18:2_16:0, PE 16:0_20:4, PE 16:0_18:2, kynurenate	**COVID + vs COVID-**<br>LPC 0:0/18:0, LPC 20:4/0:0, PC 38:6, LPC 18:2/0:0, PC 20:4_20:4, LPC 20:2/0:0, LPC 0:0/16:0, PC 18:2_22:6, LPC 20:3/0:0, LPC 18:0/0:0, LPC 17:0/0:0, LPC 14:0/0:0, LPC 16:1/0:0, LPC 16:0/0:0, LPC 18:1/0:0.	LC-MS, triple quadruple mass spectrometer, Untargeted, PCA,	339	Sindelar M., et al. [5]
6. Metabolite profile of COVID-19 revealed by UPLC-MS/MS-based widely targeted metabolomics	5-Methyluridine	N6-methyladenosine, deoxyguanosine 5'-monophosphate (dGMP), adenosine 5'-monophosphate. 11,12-EET, 2'-O-methyladenosine, 2-3-phosphate glyceride, 3'-Aenylic acid, O-phophorylethanolamine, Sn-Glycero-3-phosphocholine, 2-Aminoethanesulfonic-Acid, 9-HETE, 5-HETE, 3-Methylsalicylic acid, L-lactic acid, tryptophan, glutamic acid,12-HETE, 5,6-DiHETrE, A-Ketoglutaric Acid, Nicotinamide	UPLC-MS/MS, Targeted, PCA, OPLS-DA	128	Liu J, et al. [6]
7. Metabolomics analysis identifies glutamic acid and cystine imbalances in COVID-19 patients	Glutamic acid and serine	Glutamine, cystine, threonic acid, and proline	GC-MS, Untargeted, PCA, PLS-DA	38	Paez-Franco JC., et al. [7]

without comorbid conditions. Implications on redox homeostasis and COVID-19 pathophysiology					
8. Metabolomics analysis reveals a modified amino acid metabolism that correlates with altered oxygen homeostasis in COVID-19 patients	α-Ketoglutarate, phenylalanine, glutamic acid, and intermediaries of amino acid catabolism (3-Hydroxyisovaleric, 3-Hydroxybutyric acid, α-Hydroxybutyric acid, α-Hydroxyisovaleric, 2,3-Dihydroxybutanoic acid), α-hydroxyisovaleric acid, α-hydroxybutyric acid, 2,3 dihydroxybutanoic acid, malic acid, glutamic acid, and phenylalanine.	threonine, citrate, cysteine, isoleucine, glutamine, glyceric acid, and citric acid	UHPLC, Untargeted, PLS-DA	92	Paez-Franco JC., et al [8]
9. Metabolomics of exhaled breath in critically ill COVID-19 patients: A pilot study	**COVID-19 ARDS vs non-COVID19 ARDS** methylpent-2-enal, 2,4-octadiene, 1-chloroheptane, and nonanal.		proton-transfer reaction quadrupole time-of-flight mass spectrometer, Untargeted, PCA, OPLS-DA.	40	Grassin-Delyle S, et al. [9]
10. Metabolomics study of COVID-19 patients in four different clinical stages	Acylcarnitines (in certain patient groups), 3-hydroxybutyric acid, UFAs (like nervonic acid, linoleic acid, alpha-linolenic acid, trans-vaccenic acid, and palmitoleic acid), LPEs (16:0 and 22:6), kynurenic acid, bile acids (like taurocholic acid, taurodeoxycholic acid, glycodeoxycholic acid, glycocholic, and	Acylcarnitines (based on some previous contradictory results), LPC compounds (LPC 14:0, 0:0/16:2(omega-6), LPC 16:1, LPC 18:2, and LPC 22:6), and tryptophan.	RP/HPLC-qTOF MS/MS, Untargeted, PCA and PLS-DA	145	Valdes A, et al. [10]

	glycoursodeoxycholic acid), and purine metabolites like urea and xanthine in severe stages.				
11. Plasma metabolomics and gene regulatory networks analysis reveal the role of nonstructural SARS-CoV-2 viral proteins in metabolic dysregulation in COVID-19 patients	Sphingolipids, phospholipids, acylcarnitines, and bile acids		LC-MS/MS, Targeted, Welch's t-test.	49	Ivanisenko VA, et al. [11]
12. Proteomics and metabonomics analyses of Covid-19 complications in patients with pulmonary fibrosis	**COVID-19 with pulmonary compared to COVID-19 without pulmonary fibrosis.** 102 positive compounds upregulated while 38 negative compounds were upregulated.	**COVID-19 with pulmonary compared to COVID-19 without pulmonary fibrosis.** 21 positive compounds downregulated and 3 negative compounds downregulated	LC-MS, Untargeted	85	Yang J, et al. [12]
13. Sera Metabolomics Characterization of Patients at Different Stages in Wuhan Identifies Critical	Succinate, D-Ornithine, CRS-associated cytokines like some interleukins and interferons, 2-Ketobutyric acid, Valproic acid glucuronide, Benzoic acid, D-Glucuronic acid, Diethyl phthalic acid, PC(22:5(7Z,10Z,13Z,16Z,19Z)/18:3(6Z,9Z,12Z)), PC(22:6(4Z,7Z,10Z,13Z,16Z,19Z)/20:5(5Z,8Z,11Z,14Z,17Z)),	Several glycerophospholipids, glutamine, urea, citrulline, kynurenine inosine, Niacinamide, Propylparaben, Pyrohyperforin, Sedoheptulose, 5-Hydroperoxyeicosatetraenoic acid, 5Z-Dodecenoic acid, 9 Decenoic acid,	UHPLC, Targeted and untargeted, PCA, OPLSDA, PLSDA	199	Gu M., et al. [13]

Biomarkers of COVID-19	Phytosphingosine, Sphinganine, Styrene, Rotundine A, 2-Methylhexanoyl-CoA, 2-Pyrrolidinone, 3-(1-Pyrrolidinyl)-2-pentanone, (E)-8-(3,6-Dimethyl-2-heptenyl)-4',5,7-trihydroxyflavanone, 1,2-Benzisothiazol-3(2H)-one, 1-Arachidonoylglycerophosphoinositol, 1-Ethyl-1H-pyrrole-2-carboxaldehyde, chavicol, 1-Cyano-2-hydroxy-3-butene	Diethanolamine, Dihydrothymine, Dodecanoylcarnitine, Triethanolamine, PE(16:0/22:6(4Z,7Z,10Z,13Z,16Z,19Z)), 2-(3,4-Dihydroxybenzoyloxy)-4,6-dihydroxybenzoate, 16(17)-EpDPE, 1,2-Di-(9Z,12Z-octadecadienoyl)-sn-glycero-3-phosphate			
14. Towards risk stratification and prediction of disease severity and mortality in COVID-19: Next generation metabolomics for the measurement of host response to COVID-19 infection		Tryptophan	ESI-MS/MS, Targeted, Monte Carlo cross validation, PLS-DA, Random Forests, logistic regression models.	82	D'Amora P., et al. [14]
15. Untargeted metabolomics of COVID-19 patient serum reveals potential prognostic markers of both severity and outcome	**Deceased vs Discharged**<br>Ureidopropionate, butyrylcarnitine, 3-hydroxybutyrylcarnitine, 180.05336@4.775 kynurenic acid, deoxycytidine, adenosylhomocysteine, hexanoylcarnitine, propionylcarnitine, N1-acetylspermidine, L-octanoylcarnitine, kynurenine, creatinine, cortisol, nicotinamide	**Deceased vs Discharged**<br>Pseudouridine, uridine, arginine, tryptophan, uracil.	UHPLC-MS/MS, Untargeted, t-test, ANOVA, multiple predictor bayesian logistic regression model	120	Roberts I., et al. [15]
16. Untargeted saliva metabolomics by liquid chromatography—		Leucine, phenylalanine, tyrosine.	UHPLC, Untargeted, PCA, PLS-DA	72	Frampas CF., et al. [16]

Mass spectrometry reveals markers of COVID-19 severity					
17. Urine metabolomics links dysregulation of the tryptophan-kynurenine pathway to inflammation and severity of COVID-19	kynurenine, 3-hydroxykynurenine, and 3-hydroxyanthranilate, proline, leucine, phenylalanine, 1/2cystine, 3-methylhistidine, glutamate, asparagine, tryptophan.		LC-MS/MS, Targeted, t-test, Mann–Whitney U test, Kruskal–Wallis, FDR	56	Dewulf JP., et al. [17]
18. Biological and Clinical Factors Contributing to the Metabolic Heterogeneity of Hospitalized Patients with and without COVID-19	Proline, polyamine, citrulline, tryptophan, kynurenine, dimethylglycine, 5-OH-indoleacetate,urate	Guanidinoacetate, citrulline, proline, glutamyl-taurine, acylcarnitine C16:0, alanine, hydroxyproline, acylcarnitine C18:2, glutamyl-alanine, allantoate	UHPLC, Untargeted, PCA,PLS-DA	831	D'Alessandro A., et al. [18]
19.COVID-19 outcome prediction by integrating clinical and metabolic data using machine learning algorithms	Trimethylamine N-oxide (TMAO)		LC-MS/MS, DI-MS/MS, Targeted, PCA, PLS-DA	154	Villagrana-Banuelos KE., [19]
20. Circulating pyruvate is a potent prognostic marker for	Pyruvate, succinate, lactate, and a-ketoglutarate.		GC-qTOF, semi-targeted, Kolmogorov Smirnov test, Chi-squared	671	Ceperuelo-Mallafre V., et al. [13]

critical COVID-19 outcomes			test, Kruskall wallis test		
21. Combining Semi-Targeted Metabolomics and Machine Learning to Identify Metabolic Alterations in the Serum and Urine of Hospitalized Patients with COVID-19	pentose and glucuronate interconversion, nucleotide sugars, ascorbate, and aldarate; increased metabolites involved in cysteine, methionine, alanine, aspartate, glycine, serine, phenylalanine, tryptophan, tyrosine, valine, and leucine biosynthesis in COVID-19 patients compared to controls and non-COVID-19 patients.	glycolysis, tricarboxylic acid cycle, and biosynthesis of purine, pyrimidine, and phenylalanine	GC-qTOF, Targeted, Wilcoxon rank, FDR,	221	Baiges-Gaya G, et al. [3]
22. Distinct Metabolic Profile Associated with a Fatal Outcome in COVID-19 Patients during the Early Epidemic in Italy	3-hydroxysebacate, 5-dodecenate, tetradecadienoate, and myristoleate.<br>**Non-survivors vs survivors:** lactate, cAMP		GC-qTOF, Untargeted, univariate analysis	75	Saccon E., et al. [20]
23. Immunometabolic signatures predict risk of progression to sepsis in COVID-19.	kynurenine, kynurenine: tryptophan ratio, phenylalanine, propionic acid, beta-hydroxybutyric acid, alpha-ketoglutarate, alpha aminoadipic acid, one acylcarnitine (C10:1), and one lysophosphatidylcholine (LysoPC a 26:0) .	lysine, PC aa 36:0, PC aa 32:2, PC aa 36:6, LysoPC a 14:0, LysoPC a 16:0, LysoPC a 16:1, LysoPC a 17:0, LysoPC a 18:0, LysoPC a 18:1, LysoPC a 18:2, and SM C (20:2)	LC-MS/MS, DI-MS/MS, Targeted, PLS-DA	158	Herrera-Van Oostdam AS., et al. [21]
24.Immunomodulatory fecal metabolites are associated with mortality in COVID-19 patients with respiratory failure	**Alive compared to deceased:**<br>indole-3-carboxaldehyde, desaminotyrosine, deoxycholic acid, lithocholic acid, isodeoxycholic acid.	**Deceased compare to alive:**<br>Toluate, 3-oxodeoxycholic acid, allolithocholic acid	([–]CI-GC-MS) and ([–]LC-ESI-QTOF-MS), Targeted, Wilcoxon rank-sum test, chi-squared test,	71	Stutz MR., et al. [22]

			kaplan-meier curves.		
25. Integrative metabolomic and proteomic signatures define clinical outcomes in severe COVID-19	1-Methyladenosine, L-phenylalanine, caprylic acid, arachidonic acid,<br><br>**Associated with death**<br>Hexanoylcarnitine, L-kynurenine, cis-aconitic acid, creatine, cytosine, argininosuccinic acid.	N-acetyl-L-aspartic acid, N-acetylaspartylglutamic acid, argininosuccinic acid, ureidosuccinic acid, S-adenosylmethionine, adenosine, hypoxanthine<br>**Associated with death**<br>L-arginine, L-proline,<br><br>Targeted	LC-MS, Targeted, Linear mixed effect model	427	Buyukozkan M., et al. [23]
26. Large-Scale Plasma Analysis Revealed New Mechanisms and Molecules Associated with the Host Response to SARS-CoV-2	Several free fatty acids (FA 18:1, FA 18:2, FA 22:6, FA 44:5, FA 20:4), and unsaturated fatty acids like arachidonic, oleic, palmitic acid, and stearic acids	glycerophosphoethanolamines	UPLC-MS/MS) for lipidomics and (GCxGC-MS) for metabolomics, untargeted, PLS-DA	161	Barberis E., et al. [24]
27. Metabolic Profiling at COVID-19 Onset Shows Disease Severity and Sex-Specific Dysregulation	Linoleic acid, vaccenic acid, fumaric acid, oleic acid, d-glucarate, palmitoleic acid, 1-4 butanediol, myo-inositol, 2-hydroxybutiric acid, (E)-2-butenedioic acid, 2-hydroxyisovaleric acid, palmitic acid, (R)-3-hydroxybutiric acid, phosphoric acid, erythritol, oxoglutaric acid, myristic acid, glyceric acid, pyroglutamic acid, malic acid, glycolic acid, xanthotoxin, 1.2.4-Butanetriol, pyruvic acid, monosaccharides D, stearic acid, l-lactic acid, 2.3-butanediol, iminodiacetic acid, N-(2-acetamido)iminodiacetic acid, l-valine, monosaccharides G, methyl stearate, 2,3-dyphosphoglyceric acid, glyceraldehyde, monosaccharides C, citric acid, monosaccharides F, monosaccharides B, 2-ketoisocaproic acid, L-cysteine, monosaccharides A, 2-aminobutanoic acid.	Butylbenzene, 1,2-Dihydroxy-cyclohexene, valeramide, butanoic acid, monostearin, glycerol monostereate.	GC and CE-MS, untargeted , Chi square test, kruskal-wallis test	138	Ceballos FC., et al. [25]
28. Metabolic Reprogramming in SARS-CoV-2 Infection	Phenylalanine, ornithine, glycine, C4	Citrulline and proline, Alanine and succinylacetone, C14OH	DI-MS/MS and ESI-MS/MS, Targeted, Chi square test,	453	Martinez-Gomez LE., et al. [10]

Impacts the Outcome of COVID-19 Patients			kruskal-wallis test, univariate and bivariate exploratory analyses.		
29. Metabolic Signatures Associated with Severity in Hospitalized COVID-19 Patients	**Moderate vs Severe/critical**<br>Tryptophan	**Moderate vs Severe/critical**<br>Cer C16:0, Cer C18:0, Cer C20:0, Cer C22:0, Cer C24:1 kynurenine,3-hydroxykynurenine<br><br>Targeted	LC-MS/MS, Targeted, Chi square test, ANOVA, Bonferronis post-hoc	49	Marin-Corral J., et al. [26]
30. Metabolic Signatures of Type 2 Diabetes Mellitus and Hypertension in COVID-19 Patients with Different Disease Severity		Tryacylglicerols in diabetic patients<br>**Severe vs mild**<br>TG:22:5_34:3, TG:16:0_40:7, TG:16:0_38:6 TG:22:6_34:3 TG:16:0_38:7 TG:22:6_34:2 TG:22:6_34:2 TG:18:1_38:6 TG:22:5_32:1 TG:22:6_34:1 TG:16:0_40:8 TG:18:0_34:3 TG:18:0_38:6 TG:22:6_32:0 TG:22:5_34:2 TG:20:5_34:2, TG:22:6_32:1<br><br>Targeted	LC-MS/MS, and FIA-MS/MS for quantifying lipids, OPLS-DA	115	Elrayes MA., et al. [27]
31. Metabolite, protein, and tissue dysfunction associated with COVID-19 disease severity	cytosine, benzoate, inosine, beta-alanine, S-carboxyethylcysteine, pregnenetriol disulfate, pregnen-diol disulfate, mannose, 2S,3R-dihydroxybutyrate, 8-hydroxyoctanoate, 4-chlorobenzoic acid, N-palmitoyl-sphingosine (d18:1/16:0), 3,4 dihydroxybutyrate, N-formylphenylalanine	citruline, isoleucine, o-cresol sulfate, alpha-tocopherol, 1-arachidonoyl-GPA (20:4), tryptophan betaine, sphingosine 1-phosphate, uridine,	UPLC-MS/MS, not specified, k-nearest neighbors (KNN), Random Forest (RF), and Logistic Regression (LR)	118	Rahnavard A., et al. [28]

32. Metabolomic Profiling of Plasma Reveals Differential Disease Severity Markers in COVID-19 Patients	**Severe group vs non-severe group**<br>LysoPE(20:3) +K, Protoporphyrinogen IX +H, Deoxyguanosine and/or Adenosine +H, PS(17:0) +K, Dihydroxypalmitic acid +K, Docosahexaenyl Serotonin +K, Gamma-glutamyl-valine +Na, Isovalerylglutamic acid +Na, PI(40:5) +NH4, PS(20:4) +H-H2O, PI(44:5) +NH4, MG(18:1) +Na, SM(35:1) +K, FMC-5(38:1) +K	**Severe group vs non-severe group**<br><br>LysoPC(18:1) +H, Hydroxyoctadecenoic acid +K, PC(O-32:0) +Na, Hydroxycortisol +H-2H2Q, TG(50:3) +K, LysoPC(20:4) +H, LysoPC(16:0) +H, PAF C-16 and/or LysoPC(18:0) +H, CE(16:0) +Na, LysoPE(16:1) +H-H2O, LysoPC(16:0) +Na, CE(20:5) +H, CE(18:2) +K, PC (38:3), TG(52:5) +Na, Arachidonoyl PAF C-16 +Na, CE(16:1) +Na, PC (38:5)+Na, LysoPC(18:2) +H, LysoPC(16:0) +K, CE(18:2) +NH4, TG(52:6) +K, PAF C-16 and/or LysoPC(18:0) +Na, TG (50:4) +Na, LysoPC(16:1) +H and/or Cervonyl carnitine + Na+, PE(P-36:2)+Na, LysoPC (18:2) +Na, CE(18:3)+Na	DI and High-Electrospray Ionization Quadrupole-Orbitrap, Untargeted, Student's t-test, Wilcoxon test, Chi-square test, Fisher's exact test, univariate logistic regression, and multivariate binary logistic regression	242	Oliveira LB., et al. [29]
33. Metabolomic differences between COVID-19 and H1N1 influenza induced ARDS	**COVID-19 ARDS vs Influenza A Pneumonia**<br>free fatty acids, acetone, creatinine, and lactate	**COVID-19 ARDS vs Influenza A Pneumonia**<br>valine, 2-hydroxybutyrate, proline, methyl-guanidine, glucose, and tyrosine	NMR, Untargeted, PCA, PLS-DA.		Lorente JA., et al. [30]
34. New Insights into the Identification of Metabolites and Cytokines Predictive of Outcome for Patients with Severe SARS-CoV-2 Infection Showed Similarity with Cancer	**SARS-CoV-2 vs healthy control**<br>Creatinine, phenylalanine, 3-hydroxybutyrate, glucose, leucine, lactate.	**SARS-CoV-2 vs healthy control**<br>glutamine, glycine, and sarcosine	1H-NMR, Untargeted, Sparse Partial-Least-Squares Discriminant Analysis (sPLS-DA), Mann-Whitney U test, Receiver Operating	72	Costantini S., et al. [31]

			Characteristic (ROC) curves, and Cox regression model		
35. Plasma Metabolome Alterations Discriminate between COVID-19 and Non-COVID-19 Pneumonia	aspartic acid, glycine, serine, lactic acid, taurine, pyroglutamic acid, phenylalanine, ornithine, galactose, lyxose, maltose, ribose.	fumaric acid, n-carboxyglycine,	multiplex cytokine array from Myriad, GC-MS, untargeted, PCA, PLS-DA	92	More TH., et al. [32]
36. Predictive Biomarkers of Intensive Care Unit and Mechanical Ventilation Duration in Critically-Ill Coronavirus Disease 2019 Patients	**Invasive mechanical ventilation patients**<br>**Short (≤14 days) vs long length of stay (>14 days)**<br>Hypoxantine, Betaine<br><br>**Predictors for the duration of mechanical ventilation (**increased with longer intubation)<br>Kynurenine, p.Cresol.SO4	**Invasive mechanical ventilation patients**<br>the following metabolites were found to decrease with longer intubation: creatinine, 3-methylhistidine, Lysophosphatidylcholine C20:4 (LysoPC.a.C20.4), ornithine, sphingomyelin C24:1	FIA–MS/MS, LC–MS/MS, Targeted, OPLS-DA, univariate analysis	39	Taleb S., et al. [33]
37. Profiling metabolites and lipoproteins in COMETA, an Italian cohort of COVID-19 patients.	**COVID-19 ≤ 21 group vs post COVID19** phenylalanine, mannose, glycoproteins, isoleucine, 3-hydroxybutyric acid, acetoacetic acid, acetone, methionine, tyrosine, valine, lactic acid, acetoacetic acid, creatine, methionine,	**COVID-19 ≤ 21 group vs post COVID19**<br>Citrate, acetic acid , succininc acid, histidine, ornithine, glutamine, alanine, glycine	1H NMR, Untargeted, PCA, Random Forest algorithm, Wilcoxon-Mann-Whitney	369	Chini V., et al. [34]
38. Severe COVID-19 Is Characterised by Perturbations in	**ICU vs ward patients:** Kynurenine/Tryptophan (Kyn/Trp) ratio, kynurenine, Methionine sulfoxne, N6,N6,N6-Trimethyllysine, 4-Hydroxyproline, Glycylglycine, Glutamate, and Proline	**ICU vs ward patients:**<br>S-methylcysteine, Tryptophan, glutamine, glutathione, AABA, serine, carnosine.	LC-MS/MS, Targeted, paired t-tests for paired	44	Karu N., et al. [35]

Plasma Amines Correlated with Immune Response Markers, and Linked to Inflammation and Oxidative Stress			analyses, and Pearson correlation analyses, Benjamini–Hochberg method		
39. The Ability to Normalise Energy Metabolism in Advanced COVID-19 Disease Seems to Be One of the Key Factors Determining the Disease Progression—A Metabolomic NMR Study on Blood Plasma	**Group A (survivors) and B (negative outcome) vs healthy controls**<br>Glucose, 3-OH-butyrate, citrate, leucine, isoleucine, valine, ketoleucine, ketoisoleucine, ketovaline, creatine, phenylalanine, Phe/Tyr ratio, lysine.	**Group A and B vs healthy controls**<br>alanine, glutamine, lipoproteins, acetate.	NMR, Targeted, PCA, PLS-DA, ANOVA,Mann–Whitney U-test	108	Baranovicova E., et al. [36]
40. The Serum Metabolome of Moderate and Severe COVID-19 Patients Reflects Possible Liver Alterations Involving Carbon and Nitrogen Metabolism	lactic acid, glutamate, aspartate, phenylalanine, β-alanine, ornithine, arachidonic acid, choline, and xanthine, C18:1-OH, hypoxantine, putrescine, alanine, c4-OH-Pro, taurocholic acid, TMAO, spermine, histamine.	tiglylcarnitine (C5:1), trigonelline, hippuric acid, deoxycholic acid. In severe cases: serotonin and DHEAS (dehydroepiandrosterone sulfate), arachidonic acid, spermidine, C2, tyrosine, ornithine, C12-DC, succinic acid, citrulline, glutamine.	HPLC-MS/MS, LC-MS/MS, FIA-MS/MS, Targeted, PLS-DA	61	Caterino M. et al. [37]
41. Untargeted plasma metabolomic fingerprinting highlights several biomarkers for the	**COVID-19 vs healthy control**<br>PC(16:0/22:6(4Z,7Z,10Z,13Z,16Z,19Z)), PC(16:0/22:5(4Z,7Z,10Z,13Z,16Z)), PC(14:1(9Z)/24:1(15Z)), PC(14:0/22:4(7Z,10Z,13Z,16Z)), N-(1-Deoxy-1-fructosyl)leucine, SM(d18:0/16:1(9Z)), PC(16:0/18:2(9Z,12Z)),	**COVID-19 vs healthy control**<br>PC(14:1(9Z)/16:0), Cer(d18:1/24:1(15Z)), PC(14:0/18:2(9Z,12Z)), gamma-Glutamyltyrosine,	HPLC-MS/MS, HESI III, Untargeted, PLS-DA	110	Ocelli C., et al. [38]

diagnosis and prognosis of coronavirus disease 19.	PC(18:0/22:6(4Z,7Z,10Z,13Z,16Z,19Z)), N-(1-Deoxy-1-fructosyl)valine	PC(15:0/20:1(11Z)), Homovanillic acid sulfate			
42. Urine-based multi-omic comparative analysis of COVID-19 and bacterial sepsis-induced ARDS	**COVID 19 ARDS vs Bacterial sepsis-induced ARDS** Branched-chain aminoacids, benzoate, methionine, arginine and proline, pyrimidine,uracil, tyrosine, histidine, nicotinate and nicotinamide, ascorbate and aldarate, lysine, tryptophan, FA (Acyl carnitine, medium chain)		UPLC-MS/MS, untargeted, volcano plot	59	Batra R., et al. [39]

SUPPLEMENTAL TABLE 3. Metabolites Altered in 27 Articles Comparing Mild to Moderate/Severe COVID-19 cases.

Study (sample type)	Metabolites upregulated		Metabolites downregulated		Pathways altered	Reference
	Mild	Moderate/ Severe	Mild	Moderate/ Severe		
1. 1H qNMR-Based Metabolomics Discrimination of Covid-19 Severity	Threonine, pyruvate, alanine, citrate,tryptophan, valine.	Formate, 3-aminoisobutyrate,acetate.	Formate, 3-aminoisobutyrate,acetate	Threonine, pyruvate, alanine,citrate,tryptophan, valine.	1. Phenylalanine, tyrosine, and tryptophan biosynthesis. 2. phenylalanine metabolism 3. Pyruvate metabolism. 4. Glycerophospholipid metabolism. 5. Citrate cycle (TCA cycle) 6. Glycolysis/gluconeogénesis	Correia B.S et. al. [1]

2. Targeted metabolomics identifies high performing diagnostic and prognostic biomarkers for COVID19	Pyruvic acid, aspartic acid, hippuric acid, citric acid.	C10:2, C5, butyric acid, putrescine, glutamic acid, methionine-sulfoxide, glucose, PC ae C36:0, isoleucine, C10	C10:2, C5, butyric acid, putrescine, glutamic acid, methionine-sulfoxide, glucose, PC ae C36:0, isoleucine, C10	Pyruvic acid, aspartic acid, hippuric acid, citric acid.<br><br>LysoPC a C280 differentiates moderate from severe being downregulated in moderate cases.	No pathway analysis was done in this study.	Lopez-Hernandez Y, et al. [2]
5. Longitudinal metabolomics of human plasma reveals prognostic markers of COVID-19 disease severity.	LPC 18:1/0:0, LPC 16:0/0:0, LPC 16:1/0:0, LPC 14:0/0:0, LPC 17:0/0:0, LPC 18:0/0:0, LPC 20:3/0:0, serine, PC 18:2_22:6, LPC 0:0/16:0, LPC 20:2/0:0, PC20:4_20:4, LPC 18:2/0:0, PC 38:6, LPC 20:4/0:0, LPC 0:0/18:0	Kynurenate, PE 16:0-18:2, PE 16:0-20:4, Cer-NS d18:2-16:0, Cer NS d18:1_16:0, 1-Methyladenosine.	Kynurenate, PE 16:0-18:2, PE 16:0-20:4, Cer-NS d18:2-16:0, Cer NS d18:1_16:0, 1-Methyladenosine	LPC 18:1/0:0, LPC 16:0/0:0, LPC 16:1/0:0, LPC 14:0/0:0, LPC 17:0/0:0, LPC 18:0/0:0, LPC 20:3/0:0, serine, PC 18:2_22:6, LPC 0:0/16:0, LPC 0:0/16:0, LPC 20:2/0:0, PC20:4_20:4, LPC 18:2/0:0, PC 38:6, LPC 20:4/0:0, LPC 0:0/18:0	No pathway analysis was done in this study	Sindelar M., et al. [5]
7.Metabolomics analysis identifies glutamic acid and cystine imbalances in COVID-19 patients without comorbid conditions. Implications on redox homeostasis and COVID-19 pathophysiology	In mild/moderate patients: Glutamine 3TMS, Tyrosine 3TMS, Cholesterol, 3-Hydroxybutanoic, Palmitoleic acid, Linoleic acid, Lysine 3TMS, Leucine 2TMS, Isoleucine 2TMS,	In severe patients: glutamic acid, phenylalanine, ornithine, alpha-hydroxybutyric acid, aminobutyric acid, palmitoleic acid.	In mild/moderate patients: threonic acid, Cystine, uric acid, glycerol phosphate, 1,5-Anhydroglucito, Proline 2TMS, 4-Hydroxyproline, Threonine 3TMS, Glycerol, alpha-Hydroxybutyric acid, Aminobutyric acid,	In severe patients: threonic acid, glutamine, cystine, cholesterol, proline, tyrosine, uric acid, glycerol phosphate.	Enriched metabolite data sets ($p<0.05$) for the VIP metabolites involved in COVID-19 severity clustering: 1.D-glutamate, and D-glutamine metabolism. 2. Phenylalanine, tyrosine and tryptophan biosynthesis. 3. Arginine biosynthesis. 4. Aminoacyl-tRNA biosynthesis	Paez-Franco JC., et al. [7]

	Methionine 2TMS, and Phenylalanine 2TMS.		alpha-Hydroxyisova, citric acid, ornithine 4TMS, N-acetyl-L-lysine, Serine 3TMS, glutamic acid 3TMS, and myo-inositol			
8.Metabolomics analysis reveals a modified amino acid metabolism that correlates with altered oxygen homeostasis in COVID-19 patients	Glyceric acid, 2,4 pyrimidinedione, stearic acid, anhydroglucitol, malic acid, cysteine, citric acid, pyroglutamic acid, threonic acid, decanoic acid, alpha-ketoglutarate.	Leucine, isoleucine, alpha-hydroxyisovaleric acid, 2-aminobutanoic acid.	Leucine, isoleucine, alpha-hydroxyisovaleric acid, 2-aminobutanoic acid.	Glyceric acid, 2,4 pyrimidinedione, stearic acid, anhydroglucitol, malic acid, cysteine, citric acid, pyroglutamic acid, threonic acid, decanoic acid, alpha-ketoglutarate.	Enriched pathways employing VIP values from PLS-DA analysis differentiating mild vs severe cases:<br>1.Valine, leucine, and isoleucine degradation. 2. Citric acid cycle. 3.Glutathione metabolism. 4. Transfer of acetyl groups into mitochondria. 5.Warburg effect. 6.Glycine and serine metabolism. 6. Cysteine metabolism.	Paez-Franco JC., et al [8]
10.Metabolomics study of COVID-19 patients in four different clinical stages	7H-purine-7-acetamide, xanthine.	Urea, midazolam, hydroxychloroquine, ritonavir, lopinavir, urobilin, creatine, 1(2H)-Pyrimidineacetamide,1-methyladenosine, azithromycin, LPE (16:0), 2-dodecoxyethyl hydrogen sulfate, kynurenic acid, 4-(undecane-5-yl)benzene-1-sulfonic acid, 3 hydroxyphenylacetic acid, taurocholic acid.	Urea, midazolam, hydroxychloroquine, ritonavir, lopinavir, urobilin, creatine, 1(2H)-Pyrimidineacetamide,1-methyladenosine, azithromycin, LPE (16:0), 2-dodecoxyethyl hydrogen sulfate, kynurenic acid, 4-(undecane-5-yl)benzene-1-sulfonic acid, 3 hydroxyphenylacetic acid, taurocholic acid.	7H-purine-7-acetamide, xanthine.	1.Phenylalanine metabolism. 2. Epithelial cell signaling and H.pylori infection. 3. Synthesis and degradation of ketone bodies. 4. Biosynthesis of unsaturated fatty acids. 5.Purine metabolism. 6.Caffeine metabolism. 7. Alpha-linolenic acid metabolism. 8. Linolenic acid metabolism	Valdes A, et al. [10]
13.Sera Metabolomics Characterization of Patients at Different	1,2-Di-(9Z,12Z-octadecadienoyl)-sn-glycero-3-phosphate,	2-Ketobutyric acid, Dodecanoylcarnitine, Inosine	2-Ketobutyric acid, Dodecanoylcarnitine, Inosine	1,2-Di-(9Z,12Z-octadecadienoyl)-sn-glycero-3-phosphate, 16(17)-EpDPE,	Based on 13 differential metabolites with the most significant enrichment:	Gu M., et al. [13]

Stages in Wuhan Identifies Critical Biomarkers of COVID-19	16(17)-EpDPE, Benzeneacetonitrile, Benzoic acid, Dihydrothymine, Lactapiperanol D, Phenoxyacetic acid, Traumatic acid			Benzeneacetonitrile, Benzoic acid, Dihydrothymine, Lactapiperanol D, Phenoxyacetic acid, Traumatic acid	1.D-arginine and D-ornithine metabolism. 2. Sphingolipid metabolism. 3. Ascorbate and aldarate metabolism. 4. Valine, leucine, and isoleucine biosynthesis. 5. Nicotinate and nicotinamide metabolism. 6. Pentose and glucuronate interconversions. 7. Propanoate metabolism. 8. Steroid hormone biosynthesis. 9. Inositol phosphate metabolism. 10. Purine metabolism. 11. Glycine, serine and threonine metabolism. 12. Cysteine and methionine metabolism. 12. Arachidonic acid metabolism.	
14.Towards risk stratification and prediction of disease severity and mortality in COVID-19: Next generation metabolomics for the measurement of host response to COVID-19 infection	Citrate/ornithine ratio, tryptophan, 4-OH-pro, lysoPC a C18:2, PC ae C40:1, PC ae C42:2, PC ae C42:1, PC ae C42:6, PC ae C36:5, PC ae C36:4, total PC ae, PC ae C38:5, PC ae C34:3, PC ae C36:3, PC ae C34:2.	(Ala+Asp+Glu)/PC, (ADMA/PC ae C42:1), ADMA/PC ae C36:3, ADMA/PC ae C38:5, ADMA/PC ae C36:4, Glu/PC ae 42:1, Asp/PC ae C42:1, Asp/PC ae C36:3, [IDO/(Cit/Orn)], (ADMA/Arg)/PC aa C, (ADMA/Arg)/PC ae C, (Orn/Ser)/PC aa C4, (Orn/Ser)/PC ae C4, (Orn/Ser)/PC aa C3, (Orn/Ser)/PC ae C3	(Ala+Asp+Glu)/PC, (ADMA/PC ae C42:1), ADMA/PC ae C36:3, ADMA/PC ae C38:5, ADMA/PC ae C36:4, Glu/PC ae 42:1, Asp/PC ae C42:1, Asp/PC ae C36:3, [IDO/(Cit/Orn)], (ADMA/Arg)/PC aa C, (ADMA/Arg)/PC ae C, (Orn/Ser)/PC aa C4, (Orn/Ser)/PC ae C4, (Orn/Ser)/PC aa C3, (Orn/Ser)/PC ae C3	Citrate/ornithine ratio, tryptophan, 4-OH-pro, lysoPC a C18:2, PC ae C40:1, PC ae C42:2, PC ae C42:1, PC ae C42:6, PC ae C36:5, PC ae C36:4, total PC ae, PC ae C38:5, PC ae C34:3, PC ae C36:3, PC ae C34:2.	No pathway analysis was done in this study	D'Amora P., et al. [14]
16. Untargeted saliva metabolomics by liquid chromatography—Mass spectrometry reveals markers of COVID-19 severity	**Low vs high severity** C44H74N8O16, leucine, phenylalanine, proline, tyrosine, valine.	kynurenine, 3-hydroxykynurenine, and kynurenine to tryptophan ratio (KTR)	kynurenine, 3-hydroxykynurenine, and kynurenine to tryptophan ratio (KTR)	**Low vs high severity** C44H74N8O16, leucine, phenylalanine, proline, tyrosine, valine.	No pathway analysis was done in this study	Frampas CF., et al. [16]

17. Urine metabolomics links dysregulation of the tryptophan-kynurenine pathway to inflammation and severity of COVID-19					Tryptophan-kynurenine pathway	Dewulf JP., et al. [17]
19.COVID-19 outcome prediction by integrating clinical and metabolic data using machine learning algorithms	Lyso PC a C14:0, transhydroxyproline	C10:2, methionine sulfoxide, trimethylamine n-oxide, total dimethylarginine	C10:2, methionine sulfoxide, trimethylamine n-oxide, total dimethylarginine	Lyso PC a C14:0, transhydroxyproline	No pathway analysis was done in this study	Villagrana-Banuelos KE., [19]
20.Circulating pyruvate is a potent prognostic marker for critical COVID-19 outcomes		Critical compared to mild/severe patients: Pyruvate, succinate, lactate, and a-ketoglutarate.	Mild/severe compared to critical patients: Pyruvate, succinate, lactate, and a-ketoglutarate.		No pathway analysis was done in this study	Ceperuelo-Mallafre V., et al. [13]
23.Immunometabolic signatures predict risk of progression to sepsis in COVID-19.	Pyruvic acid, trans-hydroxyproline, hippuric acid, PC aa C40:6	C10:2, butyric acid, methionine sulfoxide, C5, isoleucine, C4, glucose, phenylalanine, glutamic acid, valine, B-hydroxybutyric acid, C4OH, C10, putrescine, spermidine, methylmalonic acid.	C10:2, butyric acid, methionine sulfoxide, C5, isoleucine, C4, glucose, phenylalanine, glutamic acid, valine, B-hydroxybutyric acid, C4OH, C10, putrescine, spermidine, methylmalonic acid.	Pyruvic acid, trans-hydroxyproline, hippuric acid, PC aa C40:6	No pathway analysis was done in this study	Herrera-Van Oostdam AS., et al. [21]
26.Large-Scale Plasma Analysis Revealed New Mechanisms and Molecules Associated with the		TG 16:1_16:1_18:2, TG 16:0_18:1_24:1,and TG 24:0_18:1_18:1, oleic acid andarachidonic acid		Glycerophosphocholine and sphingomyelins	1.Gluconeogenesis. 2.Malate-aspartate shuttle. 3. Phenylalanine and tyrosine metabolism. 4. Arginine and proline metabolism. 5. Warburg effect. 6.Beta-Alanine	Barberis E., et al. [24]

Host Response to SARS-CoV-2					metabolism. 7. Histidine metabolism. 8. Aminoacylt-RNA biosynthesis. 9. Glyoxylate and dicarboxylate metabolism	
27. Metabolic Profiling at COVID-19 Onset Shows Disease Severity and Sex-Specific Dysregulation		**Severe vs moderate cases** Monosaccharide E, Monosaccharide F, Monosaccharide B, Monosaccharide C, 2-Hydroxybutyric acid, glyceric acid, 1,2,4-Butanetriol, Monosaccharide D, Monosaccharide G, Kynurenine.		**Severe vs moderate cases** Monosaccharide A, 2-3 diphosphoglyceric acid, citric acid, 2-3 butanediol, L-lactic acid, aspartic acid, trans-3-hydroxyproline, N-methyl-L-proline, L-proline, L-serine	1.Alanine, asparte, and glutamate metabolism. 2. D-glutamine and glutamate metabolism. 3. Arginine biosynthesis. 4.Histidine metabolism. 5. Glycine, serine, and threonine metabolism. 6. Aminoacyl-tRNA biosynthesis.	Ceballos FC., et al. [25]
28. Metabolic Reprogramming in SARS-CoV-2 Infection Impacts the Outcome of COVID-19 Patients	Alanine, citrulline, proline, succinylacetone	Phenylalanine,	Phenylalanine,	Alanine, citrulline, proline, succinylacetone	Phenylalanine, and tyrosine metabolic pathways, arginine and citrulline metabolic pathway.	Martinez-Gomez LE., et al. [10]
29. Metabolic Signatures Associated with Severity in Hospitalized COVID-19 Patients	**Moderate vs Severe/critical** Tryptophan	**Severe/critical vs moderate** Cer C16:0, Cer C18:0, Cer C20:0, Cer C22:0, Cer C24:1	**Moderate vs Severe/critical** Cer C16:0, Cer C18:0, Cer C20:0, Cer C22:0, Cer C24:1		No pathway analysis was done in this study	Marin-Corral J., et al. [26]
30. Metabolic Signatures of Type 2 Diabetes Mellitus and Hypertension in COVID-19 Patients with Different Disease Severity	TG:22:5_34:3, TG:16:0_40:7, TG:16:0_38:6, TG:22:6_34:3, TG:16:0_38:7, TG:22:6_34:2 TG:22:6_34:2 TG:18:1_38:6 TG:22:5_32:1			TG:22:5_34:3, TG:16:0_40:7, TG:16:0_38:6 TG:22:6_34:3 TG:16:0_38:7 TG:22:6_34:2 TG:22:6_34:2 TG:18:1_38:6 TG:22:5_32:1	**Enriched metabolite classes in terms of COVID-19 severity** Ceramides, triacylglycerols, glycerophocpholipids, long chain polyunsaturated fatty acids, glycosylceramides, fatty acids, corticosteroids, TCA cycle, carboxylic acids, secondary bile acid metabolism, indoles	Elrayes MA., et al. [27]

	TG:22:6_34:1<br>TG:16:0_40:8<br>TG:18:0_34:3<br>TG:18:0_38:6<br>TG:22:6_32:0<br>TG:22:5_34:2<br>TG:20:5_34:2,<br>TG:22:6_32:1			TG:22:6_34:1<br>TG:16:0_40:8<br>TG:18:0_34:3<br>TG:18:0_38:6<br>TG:22:6_32:0<br>TG:22:5_34:2<br>TG:20:5_34:2,<br>TG:22:6_32:1	derivatives, benzoate metabolism.	
31.Metabolite, protein, and tissue dysfunction associated with COVID-19 disease severity	Citrulline, isoleucine	Cytosine	Cytosine	Citrulline, isoleucine	**Enrichment pathway between severe group and healthy group.**<br>1)Regulation of complement cascade pathway. 2)Purine metabolism pathway. 3)Keratinization pathway.	Rahnavard A., et al. [28]
32. Metabolomic Profiling of Plasma Reveals Differential Disease Severity Markers in COVID-19 Patients	**non-severe group vs severe group**<br><br>LysoPC(18:1) +H, Hydroxyoctadecenoic acid +K, PC(O-32:0) +Na, Hydroxycortisol +H-2H2Q, TG(50:3) +K, LysoPC(20:4) +H, LysoPC(16:0) +H, PAF C-16 and/or LysoPC(18:0) +H, CE(16:0) +Na, LysoPE(16:1) +H-H2O, LysoPC(16:0)	**Severe group vs non-severe group**<br><br>LysoPE(20:3) +K, Protoporphyrinogen IX +H, Deoxyguanosine and/or Adenosine +H, PS(17:0) +K, Dihydroxypalmitic acid +K, Docosahexaenyl Serotonin +K, Gamma-glutamyl-valine +Na, Isovalerylglutamic acid +Na, PI(40:5) +NH4, PS(20:4) +H-H2O, PI(44:5) +NH4, MG(18:1) +Na, SM(35:1) +K, FMC-5(38:1) +K	**non-severe group vs severe group**<br><br>LysoPE(20:3) +K, Protoporphyrinogen IX +H, Deoxyguanosine and/or Adenosine +H, PS(17:0) +K, Dihydroxypalmitic acid +K, Docosahexaenyl Serotonin +K, Gamma-glutamyl-valine +Na, Isovalerylglutamic acid +Na, PI(40:5) +NH4, PS(20:4) +H-H2O, PI(44:5) +NH4, MG(18:1) +Na, SM(35:1) +K, FMC-5(38:1) +K	**Severe group vs non-severe group**<br><br>LysoPC(18:1) +H, Hydroxyoctadecenoic acid +K, PC(O-32:0) +Na, Hydroxycortisol +H-2H2Q, TG(50:3) +K, LysoPC(20:4) +H, LysoPC(16:0) +H, PAF C-16 and/or LysoPC(18:0) +H, CE(16:0) +Na, LysoPE(16:1) +H-H2O, LysoPC(16:0) +Na, CE(20:5) +H, CE(18:2) +K, PC (38:3), TG(52:5) +Na, Arachidonoyl PAF C-16 +Na, CE(16:1) +Na, PC (38:5)+Na, LysoPC(18:2) +H, LysoPC(16:0) +K, CE(18:2) +NH4,	Glycerophospholipid metabolism, porphyrin metabolism, glycerolipid metabolism, purine metabolism, linoleic acid metabolism, linolenic acid metabolism, arachidonic acid metabolism, steroid biosynthesis.	Oliveira LB., et al. [29]

	+Na, CE(20:5) +H, CE(18:2) +K, PC (38:3), TG(52:5) +Na, Arachidonoyl PAF C-16 +Na, CE(16:1) +Na, PC (38:5)+Na, LysoPC(18:2) +H, LysoPC(16:0) +K, CE(18:2) +NH4, TG(52:6) +K, PAF C-16 and/or LysoPC(18:0) +Na, TG (50:4) +Na, LysoPC(16:1) +H and/or Cervonyl carnitine + Na+, PE(P-36:2)+Na, LysoPC (18:2) +Na, CE(18:3)+Na			TG(52:6) +K, PAF C-16 and/or LysoPC(18:0) +Na, TG (50:4) +Na, LysoPC(16:1) +H and/or Cervonyl carnitine + Na+, PE(P-36:2)+Na, LysoPC (18:2) +Na, CE(18:3)+Na<br><br>Untargeted		
34. New Insights into the Identification of Metabolites and Cytokines Predictive of Outcome for Patients with Severe SARS-CoV-2 Infection Showed Similarity with Cancer	**Good outcome vs exitus**<br>Glutamine, sarcosine, glycine	**Exitus vs good outcome**<br>Creatinine, phenylalanine, 3-hydroxybutyrate, glucose, leucine, lactate.	**Good outcome vs exitus**<br>Creatinine, phenylalanine, 3-hydroxybutyrate, glucose, leucine, phenylalanine, lactate.	**Exitus vs good outcome**<br>Glutamine, sarcosine, glycine	**Most significant pathways involved:**<br>Glycine, serine, and threonine metabolism, glycolysis/gluconeogenesis, glyoxylate and dicarboxylate metabolism, synthesis and degradation of ketone bodies, nitrogen metabolism, valine, leucine and isoleucine metabolism.	Costantini S., et al. [31]

35. Plasma Metabolome Alterations Discriminate between COVID-19 and Non-COVID-19 Pneumonia	**Recovered vs Deceased** Threonine, RI1157.73	**Deceased vs recovered** RI1532.53, RI1150.81	**Recovered vs deceased** RI1532.53, RI1150.81	**Deceased vs recovered** Threonine, RI1157.73	**Top 5 altered metabolic pathways in the COVID-19 pneumonia (CovP) group.** 1.Arginine biosynthesis. 2. Glutathione metabolism. 3. Aminoacyl-tRNA biosynthesis 4. Pyruvate metabolism. 5. Ala, Asp, and Gln metabolism	More TH., et al. [32]
37. Profiling metabolites and lipoproteins in COMETA, an Italian cohort of COVID-19 patients.	Citric acid	Phenylalanine, mannose, glycoproteins, isoleucine	Phenylalanine, mannose, glycoproteins, isoleucine	Citric acid	No pathway analysis was done in this study.	Chini V., et al. [34]
38. Severe COVID-19 Is Characterised by Perturbations in Plasma Amines Correlated with Immune Response Markers, and Linked to Inflammation and Oxidative Stress	**Low IL-6 vs Medium and High IL-6 patients** Glycine, tryptophan	**ICU vs ward patients:** Kynurenine/Tryptophan (Kyn/Trp) ratio, kynurenine, Methionine sulfoxne, N6,N6,N6-Trimethyllysine, 4-Hydroxyproline, Glycylglycine, Glutamate, and Proline	**Low IL-6 vs Medium and High IL-6 patients** Kynurenine, phenylalanine, cystathionine	**ICU vs ward patients:** S-methylcysteine, Tryptophan, glutamine, glutathione, AABA, serine, carnosine.	No pathway analysis was done in this study	Karu N., et al. [35]
39. The Ability to Normalise Energy Metabolism in Advanced COVID-19 Disease Seems to Be One of the Key Factors Determining the Disease Progression—A Metabolomic NMR Study on Blood Plasma	**Group A (survivors) and group B (negative outcome)** Ketovaline, alanine, leucine, tyrosine, glutamine	**Group B (negative outcome) compared to group A** Glucose, 3-hydroxybutyrate, citrate, Phe/Tyr ratio, phenylalanine	**Group A (survivors) compared to group B (negative outcome)** Glucose, 3-hydroxybutyrate, citrate, Phe/Tyr ratio, phenylalanine	**Group B (negative outcome) compared to group A** Ketovaline, alanine, leucine, tyrosine, glutamine	No pathway analysis was done in this study.	Baranovicova E., et al. [36]

40. The Serum Metabolome of Moderate and Severe COVID-19 Patients Reflects Possible Liver Alterations Involving Carbon and Nitrogen Metabolism	Trigoneline, phenylalanine, arachidonic acid, B-aminobutyric acid, spermidine, C2, C5:1, tyrosine, ornithine, C12-DC, Succinic acid, citrulline, homocysteine, tryptophan, C14:1-OH, deoxycholic acid, glutamine, serotonin.	Lactic acid, glutamate, C18:1-OH, aspartate, b-alanine, hypoxanthine, putrescine, alanine, c4-OH-Pro, taurocholic ac, TMAO, glycine, palmitic acid, C3-DC	Lactic acid, glutamate, C18:1-OH, aspartate, b-alanine, hypoxanthine, putrescine, alanine, c4-OH-Pro, taurocholic ac, TMAO, glycine, palmitic acid, C3-DC	Trigoneline, phenylalanine, arachidonic acid, B-aminobutyric acid, spermidine, C2, C5:1, tyrosine, ornithine, C12-DC, Succinic acid, citrulline, homocysteine, tryptophan, C14:1-OH, deoxycholic acid, glutamine, serotonin.	**Top 5 enriched pathways:** **Mild:** 1. glycolysis/gluconeogenesis, 2.pyruvate metabolism, 3.D-glutamine and D-glutamate metabolism,4. nitrogen metabolism, 5.pyrimidine metabolism. **Moderate:** 1. glycolysis/gluconeogenesis,2. pyruvate metabolism, 3. phenylalanine, tyrosine and tryptophan metabolism, 4.phenylalanine metabolism, 5. Arginine metabolism. **Severe:** 1. Glycolisis/gluconeogenesis, 2. pyruvate metabolism,3. alanine, aspartate and glutamate metabolism, 4.D-glutamine, and D-glutamate metabolism, 5. Nitrogen metabolism	Caterino M. et al. [37]
41. Untargeted plasma metabolomic fingerprinting highlights several biomarkers for the diagnosis and prognosis of coronavirus disease 19	PC(14:0/18:1(11Z)), indole-3-propionic acid, PC(15:0/15:0), L-Dehydroascorbic acid, PC(15:0/18:1(11Z)), L-Tryptophan, Phenylalanyl-Tryptophan, Citrulline, Oxindole, L-Glutamine, Indole, Testosterone sulfate	Isoleucylproline, N-(1-Deoxy-1-fructosyl)leucine, 1-Methylguanine	Isoleucylproline, N-(1-Deoxy-1-fructosyl)leucine, 1-Methylguanine	PC(14:0/18:1(11Z)), indole-3-propionic acid, PC(15:0/15:0), L-Dehydroascorbic acid, PC(15:0/18:1(11Z)), L-Tryptophan, Phenylalanyl-Tryptophan, Citrulline, Oxindole, L-Glutamine, Indole, Testosterone sulfate	**Mild vs Moderate and Severe subjects** Galactose metabolism, Glycosylphosphatidylinositol (GPI)-anchor biosynthesis, Ubiquinone and other terpenoid-quinone biosynthesis, Sphingolipid metabolism, Steroid biosynthesis, Pantothenate and CoA biosynthesis Drug metabolism - other enzymes, Selenocompound metabolism, Pyrimidine metabolism, D-Glutamine and D-glutamate metabolism,	Ocelli C., et al. [38]

					Nitrogen metabolism, Glutathione metabolism, Propanoate metabolism, Steroid hormone biosynthesis, Alanine, aspartate and glutamate metabolism, Porphyrin and chlorophyll metabolism<br>Glycine, serine and threonine metabolism, Arginine biosynthesis, Lysine degradation, Histidine metabolism<br>Valine, leucine and isoleucine degradation, Tryptophan metabolism, Linoleic acid metabolism, beta-Alanine metabolism, Cysteine and methionine metabolism<br>Arginine and proline metabolism, Vitamin B6 metabolism<br>Biotin metabolism, Nicotinate and nicotinamide metabolism, Arachidonic acid metabolism, Aminoacyl-tRNA biosynthesis<br>Valine, leucine and isoleucine biosynthesis<br>Glycerophospholipid metabolism, Purine metabolism<br>Tyrosine metabolism, alpha-Linolenic acid metabolism	

SUPPLEMENTAL TABLE 4 Frequency of upregulated metabolites in mild and moderate to severe COVID-19 cases across studies.

Upregulated metabolites in studies with mild COVID-19 cases	Count	Upregulated metabolites in studies with moderate to severe COVID-19 cases	Count
Tryptophan	7	Phenylalanine	6
Citrulline	4	Glucose	4
Glutamine	4	Isoleucine	4
Alanine	3	Methionine sulfoxide	4
Citric acid	3	C10:2	3
Tyrosine	3	Glutamic acid	3
4-OH-pro	2	Kynurenine	3
Glycine	2	Putrescine	3
Hippuric acid	2	(ADMA/Arg)/PC aa C	2
Leucine	2	(ADMA/Arg)/PC ae C	2
LysoPC a C18:2	2	(Ala+Asp+Glu)/PC	2
PAF C-16	2	(Orn/Ser)/PC aa C3	2
PC ae C34:2	2	(Orn/Ser)/PC aa C4	2
PC ae C34:3	2	(Orn/Ser)/PC ae C3	2
PC ae C36:4	2	(Orn/Ser)/PC ae C4	2
PC ae C36:5	2	[IDO/(Cit/Orn)]	2
PC ae C38:5	2	1-Methyladenosine	2
PC ae C40:1	2	3-hydroxybutyrate	2
PC ae C42:1	2	Asp/ PC ae C36:3	2
PC ae C42:2	2	Asp/ PC ae C42:1	2
PC ae C42:6	2	Butyric acid	2
Proline	2	C10	2
Pyruvic acid	2	C5	2
TG:22:6_34:2	2	Glu/ PC ae C42:1	2

Threonine	2	Glutamate	2
Total PC ae	2	Kyn/Trp	2
Transhydroxyproline	2	Kynurenic acid	2
Valine	2	Lactate	2
		Leucine	2
		PI(40:5) +NH4	2
		Taurocholic acid	2

SUPPLEMENTAL TABLE 5: Frequency of downregulated metabolites in mild and moderate to severe COVID-19 cases across studies.

Downregulated metabolites in studies with mild COVID-19 cases	Count	Downregulated metabolites in studies with moderate to severe COVID-19 cases	Count
Phenylalanine	7	Glutamine	6
Glucose	4	Tryptophan	6
Isoleucine	4	Citric acid	4
C10:2	3	Citrulline	4
Methionine sulfoxide	3	PC ae C36:5	4
Putrescine	3	Proline	4
(ADMA/Arg)/PC aa C	2	Tyrosine	4
(ADMA/Arg)/PC ae C	2	Alanine	3
(Ala+Asp+Glu)/PC	2	Serine	3
(Orn/Ser)/PC aa C3	2	4-OH-pro	2
(Orn/Ser)/PC aa C4	2	Aspartic acid	2
(Orn/Ser)/PC ae C3	2	CE(16:0) +Na	2
(Orn/Ser)/PC ae C4	2	Cysteine	2

1-Methyladenosine	2	Hippuric acid	2
3-hydroxybutyrate	2	Leucine	2
alpha-hydroxyisovaleric acid	2	LPC 0:0/16:0	2
Asp/ PC ae C36:3	2	LysoPC a C18:2	2
Asp/ PC ae C42:1	2	PAF C-16	2
Butyric acid	2	PC ae C34:2	2
C10	2	PC ae C34:3	2
C5	2	PC ae C36:4	2
Glu/ PC ae C42:1	2	PC ae C40:1	2
Glutamic acid	2	PC ae C42:1	2
Kynurenate	2	PC ae C42:2	2
Kynurenine	2	PC ae C42:6	2
Lactate	2	Phenylalanine	2
Leucine	2	Pyruvic acid	2
PI(40:5) +NH4	2	Threonic acid	2
Taurocholic acid	2	Threonine	2
		Total PC ae	2
		Valine	2

References:

1. Correia, B. S. B., V. G. Ferreira, Pmfd Piagge, et al. (1)H Qnmr-Based Metabolomics Discrimination of Covid-19 Severity. *J Proteome Res* **2022**, 21, no. 7: 1640-53.
2. López-Hernández, Y., J. Monárrez-Espino, A. H. Oostdam, et al. Targeted Metabolomics Identifies High Performing Diagnostic and Prognostic Biomarkers for Covid-19. *Sci Rep* **2021**, 11, no. 1: 14732.
3. Baiges-Gaya, G., S. Iftimie, H. Castañé, et al. Combining Semi-Targeted Metabolomics and Machine Learning to Identify Metabolic Alterations in the Serum and Urine of Hospitalized Patients with Covid-19. *Biomolecules* **2023**, 13, no. 1.
4. Ansone, L., M. Briviba, I. Silamikelis, et al. Amino Acid Metabolism Is Significantly Altered at the Time of Admission in Hospital for Severe Covid-19 Patients: Findings from Longitudinal Targeted Metabolomics Analysis. *Microbiol Spectr* **2021**, 9, no. 3: e0033821.
5. Sindelar, M., E. Stancliffe, M. Schwaiger-Haber, et al. Longitudinal Metabolomics of Human Plasma Reveals Prognostic Markers of Covid-19 Disease Severity. *Cell Rep Med* **2021**, 2, no. 8: 100369.
6. Liu, J., Z. B. Li, Q. Q. Lu, et al. Metabolite Profile of Covid-19 Revealed by Uplc-Ms/Ms-Based Widely Targeted Metabolomics. *Front Immunol* **2022**, 13894170.
7. Páez-Franco, J. C., J. L. Maravillas-Montero, N. R. Mejía-Domínguez, et al. Metabolomics Analysis Identifies Glutamic Acid and Cystine Imbalances in Covid-19 Patients without Comorbid Conditions. Implications on Redox Homeostasis and Covid-19 Pathophysiology. *PLoS One* **2022**, 17, no. 9: e0274910.
8. Páez-Franco, J. C., J. Torres-Ruiz, V. A. Sosa-Hernández, et al. Metabolomics Analysis Reveals a Modified Amino Acid Metabolism That Correlates with Altered Oxygen Homeostasis in Covid-19 Patients. *Sci Rep* **2021**, 11, no. 1: 6350.
9. Grassin-Delyle, S., C. Roquencourt, P. Moine, et al. Metabolomics of Exhaled Breath in Critically Ill Covid-19 Patients: A Pilot Study. *EBioMedicine* **2021**, 63103154.
10. Martínez-Gómez, L. E., I. Ibarra-González, C. Fernández-Lainez, et al. Metabolic Reprogramming in Sars-Cov-2 Infection Impacts the Outcome of Covid-19 Patients. *Front Immunol* **2022**, 13936106.
11. Ivanisenko, V. A., E. V. Gaisler, N. V. Basov, et al. Plasma Metabolomics and Gene Regulatory Networks Analysis Reveal the Role of Nonstructural Sars-Cov-2 Viral Proteins in Metabolic Dysregulation in Covid-19 Patients. *Sci Rep* **2022**, 12, no. 1: 19977.
12. Yang, J., C. Chen, W. Chen, et al. Proteomics and Metabonomics Analyses of Covid-19 Complications in Patients with Pulmonary Fibrosis. *Sci Rep* **2021**, 11, no. 1: 14601.

13. Ceperuelo-Mallafré, V., L. Reverté, J. Peraire, et al. Circulating Pyruvate Is a Potent Prognostic Marker for Critical Covid-19 Outcomes. *Front Immunol* **2022**, 13912579.
14. D'Amora, P., Idcg Silva, M. A. Budib, et al. Towards Risk Stratification and Prediction of Disease Severity and Mortality in Covid-19: Next Generation Metabolomics for the Measurement of Host Response to Covid-19 Infection. *PLoS One* **2021**, 16, no. 12: e0259909.
15. Roberts, I., M. Wright Muelas, J. M. Taylor, et al. Untargeted Metabolomics of Covid-19 Patient Serum Reveals Potential Prognostic Markers of Both Severity and Outcome. *Metabolomics* **2021**, 18, no. 1: 6.
16. Frampas, C. F., K. Longman, M. Spick, et al. Untargeted Saliva Metabolomics by Liquid Chromatography-Mass Spectrometry Reveals Markers of Covid-19 Severity. *PLoS One* **2022**, 17, no. 9: e0274967.
17. Dewulf, J. P., M. Martin, S. Marie, et al. Urine Metabolomics Links Dysregulation of the Tryptophan-Kynurenine Pathway to Inflammation and Severity of Covid-19. *Sci Rep* **2022**, 12, no. 1: 9959.
18. D'Alessandro, A., T. Thomas, I. J. Akpan, et al. Biological and Clinical Factors Contributing to the Metabolic Heterogeneity of Hospitalized Patients with and without Covid-19. *Cells* **2021**, 10, no. 9.
19. Villagrana-Bañuelos, K. E., V. Maeda-Gutiérrez, V. Alcalá-Rmz, et al. Covid-19 Outcome Prediction by Integrating Clinical and Metabolic Data Using Machine Learning Algorithms. *Rev Invest Clin* **2022**, 74, no. 6: 314-27.
20. Saccon, E., A. Bandera, M. Sciumè, et al. Distinct Metabolic Profile Associated with a Fatal Outcome in Covid-19 Patients During the Early Epidemic in Italy. *Microbiol Spectr* **2021**, 9, no. 2: e0054921.
21. Herrera-Van Oostdam, A. S., J. E. Castañeda-Delgado, J. J. Oropeza-Valdez, et al. Immunometabolic Signatures Predict Risk of Progression to Sepsis in Covid-19. *PLoS One* **2021**, 16, no. 8: e0256784.
22. Stutz, M. R., N. P. Dylla, S. D. Pearson, et al. Immunomodulatory Fecal Metabolites Are Associated with Mortality in Covid-19 Patients with Respiratory Failure. *Nat Commun* **2022**, 13, no. 1: 6615.
23. Buyukozkan, M., S. Alvarez-Mulett, A. C. Racanelli, et al. Integrative Metabolomic and Proteomic Signatures Define Clinical Outcomes in Severe Covid-19. *iScience* **2022**, 25, no. 7: 104612.
24. Barberis, E., S. Timo, E. Amede, et al. Large-Scale Plasma Analysis Revealed New Mechanisms and Molecules Associated with the Host Response to Sars-Cov-2. *Int J Mol Sci* **2020**, 21, no. 22.
25. Ceballos, F. C., A. Virseda-Berdices, S. Resino, et al. Metabolic Profiling at Covid-19 Onset Shows Disease Severity and Sex-Specific Dysregulation. *Front Immunol* **2022**, 13925558.
26. Marín-Corral, J., J. Rodríguez-Morató, A. Gomez-Gomez, et al. Metabolic Signatures Associated with Severity in Hospitalized Covid-19 Patients. *Int J Mol Sci* **2021**, 22, no. 9.
27. Elrayess, M. A., F. S. Cyprian, A. M. Abdallah, et al. Metabolic Signatures of Type 2 Diabetes Mellitus and Hypertension in Covid-19 Patients with Different Disease Severity. *Front Med (Lausanne)* **2021**, 8788687.

28. Rahnavard, A., B. Mann, A. Giri, et al. Metabolite, Protein, and Tissue Dysfunction Associated with Covid-19 Disease Severity. *Sci Rep* **2022**, 12, no. 1: 12204.
29. Oliveira, L. B., V. I. Mwangi, M. A. Sartim, et al. Metabolomic Profiling of Plasma Reveals Differential Disease Severity Markers in Covid-19 Patients. *Front Microbiol* **2022**, 13844283.
30. Lorente, J. A., N. Nin, P. Villa, et al. Metabolomic Diferences between Covid-19 and H1n1 Influenza Induced Ards. *Crit Care* **2021**, 25, no. 1: 390.
31. Costantini, S., G. Madonna, E. Di Gennaro, et al. New Insights into the Identification of Metabolites and Cytokines Predictive of Outcome for Patients with Severe Sars-Cov-2 Infection Showed Similarity with Cancer. *Int J Mol Sci* **2023**, 24, no. 5.
32. More, T. H., B. Mozafari, A. Märtens, et al. Plasma Metabolome Alterations Discriminate between Covid-19 and Non-Covid-19 Pneumonia. *Metabolites* **2022**, 12, no. 11.
33. Taleb, S., H. M. Yassine, F. M. Benslimane, et al. Predictive Biomarkers of Intensive Care Unit and Mechanical Ventilation Duration in Critically-Ill Coronavirus Disease 2019 Patients. *Front Med (Lausanne)* **2021**, 8733657.
34. Ghini, V., G. Meoni, L. Pelagatti, et al. Profiling Metabolites and Lipoproteins in Cometa, an Italian Cohort of Covid-19 Patients. *PLoS Pathog* **2022**, 18, no. 4: e1010443.
35. Karu, N., A. Kindt, A. J. van Gammeren, et al. Severe Covid-19 Is Characterised by Perturbations in Plasma Amines Correlated with Immune Response Markers, and Linked to Inflammation and Oxidative Stress. *Metabolites* **2022**, 12, no. 7.
36. Baranovicova, Eva, Anna Bobcakova, Robert Vysehradsky, et al. The Ability to Normalise Energy Metabolism in Advanced Covid-19 Disease Seems to Be One of the Key Factors Determining the Disease Progression—a Metabolomic Nmr Study on Blood Plasma. *Applied Sciences* **2021**, 11, no. 9: 4231.
37. Caterino, M., M. Costanzo, R. Fedele, et al. The Serum Metabolome of Moderate and Severe Covid-19 Patients Reflects Possible Liver Alterations Involving Carbon and Nitrogen Metabolism. *Int J Mol Sci* **2021**, 22, no. 17.
38. Occelli, C., J. M. Guigonis, S. Lindenthal, et al. Untargeted Plasma Metabolomic Fingerprinting Highlights Several Biomarkers for the Diagnosis and Prognosis of Coronavirus Disease 19. *Front Med (Lausanne)* **2022**, 9995069.
39. Batra, R., R. Uni, O. M. Akchurin, et al. Urine-Based Multi-Omic Comparative Analysis of Covid-19 and Bacterial Sepsis-Induced Ards. *Mol Med* **2023**, 29, no. 1: 13.
